# Comparison of deferred and bedside culture of *Neisseria gonorrhoeae*: a study to improve the isolation of gonococci for antimicrobial susceptibility testing

**Published:** 2020-06

**Authors:** Iryna Boiko, Yuliia Stepas, Inna Krynytska

**Affiliations:** 1 Department of Functional and Laboratory Diagnostics, I. Horbachevsky Ternopil National Medical University, Ternopil, Ukraine; 2 Department of Clinical Laboratory Diagnostics, Danylo Halytskyi Lviv National Medical University, Lviv, Ukraine

**Keywords:** *Neisseria gonorrhoeae*, Culture, Transport media, Antimicrobial susceptibility

## Abstract

**Background and Objectives:**

Antimicrobial resistance of *Neisseria gonorrhoeae* is globally spread and threatening. Culturing of *N. gonorrhoeae* is the only method to collect live isolates for investigation antimicrobial resistance profile. Therefore, quality assessment of *N. gonorrhoeae* culture is essential for successful isolation of gonococci. This study was conducted to evaluate deferred and bedside culture of *N. gonorrhoeae* depending on the year season and temperature condition of transport media temporary storage.

**Materials and Methods:**

Urogenital swabs from 46 symptomatic heterosexual patients with gonorrhoea and subculture of *N. gonorrhoeae* in 46 suspensions in concentrations 1.5 × 10^8^ CFU/ml were subjected to the study. Non-nutritive transporting medium Amies Agar Gel Medium with charcoal (Copan Diagnostics Inc., Brescia, Italy) was used for deferred culture and selective Chocolate agar TM+PolyViteX VCAT3 (BioMérieux, Marcy-l’Étoile, France) for both tested methods of culture.

**Results:**

The specificity of both bedside and deferred methods of culture was 100%. The sensitivity of deferred culture was higher than of bedside culture (82.6% vs 47.8%, p<0.0005). Deferred culture showed significantly higher sensitivity comparing to bedside culture in summer (100% vs 50%, p=0.003), and comparably the same as for bedside culture in autumn, winter and spring.

**Conclusion:**

The viability of *N. gonorrhoeae* subcultures was significantly higher in refrigerated samples from transport media than from ambient one after exposition from 48 to 96 hours. Optimal viability of *N. gonorrhoeae* was observed when transport swabs were kept refrigerated up to 48 h (73.9–93.5%) or ambiently – up to 24 h (87%). Updating laboratory guidelines regarding sampling and timely specimen processing might improve gonococcal culture performance.

## INTRODUCTION

Gonorrhoea is the second most common sexually transmitted infection (STI) spreading worldwide, caused by Gram-negative diplococci *Neisseria gonorrhoeae* (1–3). It is a serious public health problem as untreated infection may lead to severe secondary sequelae, including pelvic inflammatory disease, first-trimester miscarriages, ectopic pregnancy and infertility. *N. gonorrhoeae* infections also facilitate HIV acquisition and transmission (2, 4, 5). Antimicrobial surveillance monitoring has been set up in many countries worldwide (6–8) to provide an urgent and adequate response to the exceptional ability of *N. gonorrhoeae* to develop resistance almost to all anti-microbials used in various treatment regimes. WHO recommends systematically to investigate the antimicrobial resistance profile of sufficiently large and representative of reported gonorrhoea cases in each country yearly (9). Culture remains the only routine laboratory method to provide alive gonococci for antimicrobial resistance study. Gonococci are fastidious sexually transmitted bacteria and require particular cultivation conditions to survive *in vitro* (10), which raises a challenge for culture diagnostics even nowadays (11–13). Temporary storage and transportation conditions along with the collection of biological material, are the significant part of pre-analytical microbiology process. Bedside culturing with direct and immediate inoculation of biological specimens on the agar plate is considered a golden standard for *N. gonorrhoeae* culture (10). However, this method is time-consuming, it imposes additional duties on clinicians: to keep pre-warm agar plates, to correctly distribute biological material onto the agar surface, to collocate thermostat in the examination room, to monitor the incubation conditions (temperature, humidity and CO_2_ indicators), even during short-term temporary storage before transportation to the laboratory.

The other main practical problem that confronts us is that many biological specimens are transported to the diagnostic laboratories over long distances and time intervals. Considering the obstacles, the use of transport media is recommended to maximise *N. gonorrhoeae* survival and sample quality (10). Transport medium is the most relevant for clinical departments located distantly from laboratories. The fluctuation of ambient temperature during the temporary storage and transportation plays a significant role in the further successful cultivation and identification of *N. gonorrhoeae* strains. The climate in Ukraine has wide temperature fluctuation during a year – from the highest in July (13/24°C) to the lowest in winter (0/−6°C) (https://en.m.wikipedia.org/wiki/Geography_of_Ukraine). Different climate conditions could be an issue for samples transportation, despite even the use of thermo-protected boxes. To the best of our knowledge, quality evaluation of specimen inoculation methods for *N. gonorrhoeae* culture has not been performed in Ukraine during the last 15 years, and no study has been reported internationally.

To improve isolation of *N. gonorrhoeae* for antimicrobial resistance study in Ternopil region of Ukraine, we aimed to compare two methods of *N. gonorrhoeae* culture: bedside and deferred by using transporting Amies Agar Gel Medium with charcoal (Copan Diagnostics Inc., Brescia, Italia) and selective Chocolate agarTM+PolyViteX VCAT3 media (bioMérieux, Marcy-l’Étoile, France); to assess the isolation rate of *N. gonorrhoeae* depending on annual seasons and temperature condition of transport media temporary storage.

## MATERIALS AND METHODS

### Study design.

The present comparative pilot study was conducted in two phases. Firstly, bedside and deferred culture using a non-nutritive transport Amies Agar Gel Medium with charcoal (Copan Diagnostics Inc., Brescia, Italy) with selective Chocolate agar TM+PolyViteX VCAT3 media (bioMérieux, Marcy-l’Étoile, France) were compared in isolation rate during different seasons of the year. Secondly, a non-nutritive transport medium with charcoal was assessed at different temperature regimes (ambient and refrigerated) and time of storage (0 h, 24 h, 48 h, 72 h, 96 h) basing on the preservation of the viability of *N. gonorrhoeae* subcultures.

### Patient population.

Forty-six symptomatic patients with urogenital discharges attending Ternopil Regional STI Clinic (Ukraine) from January 2016 to December 2017 were enrolled in the study. The informed consent was obtained from all participants. Diagnosis of gonorrhoea was made according to the presence of Gram-negative intracellular diplococci and leukocytosis in urogenital smears in microscopy and isolation of *N. gonorrhoeae* in culture. All isolates were cultured onto a selective agar media Chocolate agar TM+PolyViteX VCAT3 (BioMerieux Ltd, France) and subsequently confirmed as *N. gonorrhoea* by identification of Gram-negative diplococci in microscopy, rapid oxidase reaction (OXI test diagnostic strips, Microlatest; Czech Republic), and rapid sugar utilization test (Neisseria-test, PLIVA - Lachema Diagnostika s.r.o., Czech Republic). *N. gonorrhoeae* isolates were cultured as part of the standard patient management protocol, and no patient identification information was disclosed in the study. Patients received treatment of gonorrhoea and concomitant STI according to the guidelines of the Ministry of Health of Ukraine issued in 2004 and 2009 (14, 15).

### Biological specimens.

Specimens for *N. gonorrhoeae* culture were collected from male urethra and female cervix. The first specimen was promptly inoculated by sterile probe directly onto a selective Chocolate agar TM+PolyViteX VCAT3 media (bioMérieux, Marcy-l’Étoile, France) (selective chocolate agar) in the examination room and immediately placed in the thermostat for 24–48 hours at 36 ± 1°C in a humid candle jar. Second specimen was taken with the swab from non-nutritive transporting Amies Agar Gel Medium with charcoal (Copan Diagnostics Inc., Brescia, Italy) (non-nutritive transport medium), temporary stored in the examination room during 0.5–6 hours at ambient temperature 18–25°C and then transported in the thermo-protected box during 10–15 min to the Laboratory department of Ternopil Regional STI Clinic where laboratory specialist inoculated swabs from the transport media onto agar plate with selective chocolate agar. All samples were incubated for 24–48 hours at 36 ± 1°C in a humid candle jar. Isolates were confirmed as *N. gonorrhoeae* as described above.

### *Neisseria gonorrhoeae* subcultures.

Fresh subcultures of 46 clinical isolates of *N. gonorrhoeae* were cultivated on selective chocolate agar for 18–24 hours at 36 ± 1°C in a humid candle jar and confirmed as *N. gonorrhoeae*. Fresh growth from culture plates was used to prepare suspensions of each bacterium in saline equivalent to a 0.5 McFarland Standard (bioMérieux, Marcy-l’Étoile, France), approximately 1.5 × 10^8^ CFU/ml.

For each tested isolate (n=46) five swabs from the transport system were inoculated with 100 μL of the bacterial suspension for each evaluated time point (0 h, 24 h, 48 h, 72 h, 96 h). This procedure was duplicated, so results of two plate cultures for each saline dilution were counted.

### Statistical analysis.

The bedside and deferred culture of *N. gonorrhoeae* were compared for differences in isolation rates, the time required for isolation, a year season of performing diagnostics and temperate of transport media temporary storage. The results were considered positive when at least one typical colony was present and confirmed as *N. gonorrhoeae*. True positive and false negative samples, positive predictive value (PPV) and sensitivity were calculated with 95% confidence intervals (95% CI) using the exact binomial distribution method. P-value was displayed with a threshold at 0.05. Statistical analysis was performed by MedCalc Statistical Software version 18.11.3 (MedCalc Software bvba, Ostend, Belgium).

### Ethical approval.

Bioethics Commission of I. Horbachevsky Ternopil State Medical University (Ukraine) approved the study (Excerpts from Minutes No. 29, dated 20.05.2015).

## RESULTS

Samples for culture were taken from 46 symptomatic heterosexual patients, including 38 male and 8 female patients, 28.5 years old on average (range 17–80). All patients presented urogenital discharges and have been diagnosed with gonorrhoea. Both methods of culture achieved 100% positive predictive value (PPV). More than half of false-negative results (52.2%, 24/46) were obtained using bedside culture and 17.4% (8/46) – using deferred culture. No false-negative results were obtained using transport media during summer, while bedside culture provided 50% (7/14) of false-negative results. Sensitivity was significantly higher using the transport system comparing to the bedside culture (82.6% vs 47.8%, p=0.005). Deferred culture showed significantly higher sensitivity comparing to bedside culture in summer (100% vs 50%, p=0.003), and was comparably the same as bedside culture in other 3 of 4 year seasons, accordingly 83.3% vs 50% in autumn (p=0.09), 66.7% vs 55.6% in winter (p=0.64) and 72.7% vs 36.4% in spring (p=0.09). The proportion of positive gonococcal culture depending on culture methods and year seasons is shown in Table 1.

**Table 1.** The proportion of cases provided a positive gonococcal culture by season and mode of specimen inoculation onto agar pates

**Season**	**Mode of inoculation**	**True positive**			**False-negative**			**Sensitivity**		**PPV**	

		**N**	**%**	**(95%CI)**	**n**	**%**	**(95%CI)**	**%**	**(95%CI)**	**%**	**(95% CI)**
		P value^*^			p-value^*^			p-value^*^			
Winter, n=9	Directly plated	5	55.6	(21.2–86.3)	4	44.4	(13.7–78.8)	55.6	(21.2–86.3)	100	(66.4–100)
	Deferred plated	6	66.7	(29.9–92.5)	3	33.3	(7.5–70.1)	66.7	(29.9–92.5)	100	(66.4–100)
		**p=0.64**			**p=0.64**			**p=0.64**			
Spring, n=11	Directly plated	4	36.4	(10.9–69.2)	7	63.6	(30.8–89.1)	36.4	(10.9–69.2)	100	(71.5–100)
	Deferred plated	8	72.7	(39–94)	3	27.3	(6–61)	72.7	(39–94)	100	(71.5–100)
		**p=0.09**			**p=0.09**			**p=0.09**			
Summer, n=14	Directly plated	7	50	(23–77)	7	50	(23–77)	50	(23–77)	100	(76.8–100)
	Deferred plated	14	100	(76.8–100)	0	0	(0–23.2)	100	(76.8–100)	100	(76.8–100)
		**p=0.003**			**p=0.003**			**p=0.003**			
Autumn, n=12	Directly plated	6	50	(21.1–78.9)	6	50	(21.1–78.9)	50	(21.1–78.9)	100	(73.5–100)
	Deferred plated	10	83.3	(51.6–97.9)	2	16.7	(2.1–48.4)	83.3	(51.6–97.9)	100	(73.5–100)
		**p=0.09**			**p=0.09**			**p=0.09**			
All seasons, n=46	Directly plated	22	47.8	(32.9–63.1)	24	52.2	(36.9–67.1)	47.8	(32.9–63.1)	100	(92.3–100)
	Deferred plated	38	82.6	(68.6–92.2)	8	17.4	(7.8–31.4)	82.6	(68.6–92.2)	100	(92.3–100)
		**p=0.0005**			**p=0.0005**			**p=0.0005**			

PPV, positive predictive value; CI, confidence interval; p, p-value.

No significant differences in positive culture results between the ambient and refrigerated temperature regimes of temporary storage of biological specimens in the transport medium were observed in 0–24 hours of time check-points. However, positive results of *N. gonorrhoeae* cultures were received significantly more frequently from refrigerated samples than from ambient, accordingly after 48 h – in 73.9% vs 52.2% (p=0.03), after 72 h of storage in 58.7% vs 37% (p=0.04), and after 96 h – in 47.8% vs 17.4% (p=0.002). The viability of subcultures decreased rapidly in all cases from 24 h to 96 h of storage, and nonviable subcultures were obtained significantly more frequently from ambient transport media than refrigerated one after 48 h – 47.8% vs 26.1% (p=0.03), 72 h – 63% vs 41.3% (p=0.04) and 96 h – 82.6% vs 52.2% (p=0.002) respectively. The details for the viability of gonococcal subcultures in different time check-points and temperatures of temporary storage of sampled transport media are shown in Table 2.

**Table 2.** The proportion of viability of gonococcal subcultures by the different time of inoculation stored at ambient and refrigerated temperatures

**Temperature of temporary storage**	***N. gonoroeae* isolate**	**0 h**			**24 h**			**48 h**			**72 h**			**96 h**		

		**n**	**%**	**(95%CI)**	**n**	**%**	**(95%CI)**	**n**	**%**	**(95%CI)**	**n**	**%**	**(95%CI)**	**n**	**%**	**(95%CI)**
ambient (n=46)	viable	46	100	(92.3–100)	40	87	(73.8–95.1)	24	52.2	(37–67.1)	17	37	(23.2–52.5)	8	17.4	(7.8–31.4)
refrigerated (n=46)	viable	46	100	(92.3–100)	43	93.5	(82.1–98.6)	34	73.9	(58.9–85.7)	27	58.7	(43.2–73)	22	47.8	(32.9–63)
p-value			1			0.296			0.03			0.04			0.002	
ambient (n=46)	nonviable	0	0	(0–7.7)	6	13	(4.9–26.2)	22	47.8	(32.9–63)	29	63	(47.5–76.8)	38	82.6	(68.6–92.2)
refrigerated (n=46)	nonviable	0	0	(0–7.7)	3	6.5	(1.6–18.6)	12	26.1	(14.3–41.2)	19	41.3	(27–56.8)	24	52.2	(37–67.1)
p-value			0			0.296			0.03			0.04			0.002	

CI, confidence interval.

## DISCUSSION

Gonorrhoea cases have been risen worldwide over the last decades, especially in the well-developed countries (3). Use of highly sensitive and specific molecular tests and genome-based technics lead to effective screening and diagnostic efforts (16). In less-developed countries, for example, in Ukraine, microscopy and culture remain to be basic laboratory methods for screening and diagnosing gonorrhoea. Evaluation of quality performance is the crucial step for accurate laboratory testing on STI, including *N. gonorrhoeae* (17–20). Pre-analytical steps of culture play an essential role in the successful *N. gonorrhoeae* isolation (10). This study was designed to compare the ability of deferred and bedside culture to maintain the viability of *N. gonorrhoeae* depending on year seasons, time and temperature conditions of transport media temporary storage. Non-nutritive transport medium with charcoal for the deferred culture of *N. gonorrhoeae* was evaluated as a method of improving the specimen quality.

Transport media showed better performance characteristics in our study comparae to the gold standard bedside culture. Combination of using non-nutritive transport system with charcoal with further inoculation of the biologic specimens onto selective chocolate agar had optimal sensitivity compared to bedside culture (82.6% vs 47.8%, p=0.0005). The deferred culture showed higher sensitivity comparing with directly plated material in summer (100% vs 50%, p=0.003), and comparably the same sensitivity as bedside culture in autumn, winter and spring. The highest isolation rate of *N. gonorrhoeae* (98%) using deferred culture with Copan Amies gel agar with charcoal was described by Olsen et al. (21). Radebe et al. showed the viability of 62.4–88.2% for deferred culture using Amies transport medium, that is similar to the results of our study (22).

The study confirmed that refrigerated samples, which were stored in the non-nutritive transport media during 48 h, 72 h and 96 h had positive culture results more frequently than those stored at ambient condition, i.e. 73.9% vs 52.2%, 58.7% vs 37%, 47.8% vs 17.4% respectively, which is in line with Serra-Pladevall J et al. (23). The best viability rate was for refrigerated transport media during 24 h (93.5%) and 48 h (73.9%). The acceptable viability of *N. gonorrhoeae* was for transport media ambient storage during 24 h (87%). For longer than 24 h temporary ambient storage and longer than 48 h temporary refrigerated storage the viability was suboptimal: 52.2–17.4% and 58.7–47.8% respectively, which was accordingly with Farhat et al. (24). The possibility of preservation of samples for a relatively long period (within 24–48 hours) using transport media without the loss of viability of gonococci is crucial for clinical departments located distantly from the laboratory. Otherwise, when storage is needed for more than 96 h, nutritive transport media are recommended (10). Our study suggested the use of nutritive transport media for temporary storage for more than 48 h to maintain the best performance characteristics of deferred culture for *N. gonorrhoeae*.

This study changed the procedure of transportation and inoculation of specimen samples at Ternopil regional STI clinic by the implementation of Amies Agar Gel Medium with charcoal (Copan Diagnostics Inc., Italy) and selective Chocolate agar TM+Poly-ViteX VCAT3 media (bioMérieux, Marcy-l’Étoile, France) for *N. gonorrhoeae* culture. This study made it possible to collect 136 pure isolates of *N. gonorrhoeae* and investigate their antimicrobial profiles in the WHO reference laboratory for the first time in Ukraine (25).

However, our study had several limitations: this pilot study was based on the relatively small size of urogenital samples and subcultures (46) from the limited geographic population, i.e. from 1 of 24 regions of Ukraine and no reference strain of *N. gonorrhoeae* was used due to its absence in Ukraine. Despite known limitations, our pilot study might support future expanded evaluation of *N. gonorrhoeae* culture in Ukraine.

## CONCLUSION

Deferred culture of *N. gonorrhoeae* with the use of non-nutritive transport system Amies Agar Gel Medium with charcoal and selective Chocolate agar TM+PolyViteX VCAT3 (BioMérieux, Marcy-l’Étoile, France) has significantly higher reproducing quality than bedside culture with the same selective media for isolation of *N. gonorrhoeae*. Deferred culture has significantly higher sensitivity comparing to directly plated material in summer, and was comparably the same as bedside culture in autumn, in winter and spring. Storage of samples in refrigerated non-nutritive transport media with charcoal is superlative over the ambient condition and has optimal performance for exposition up to 48 hours. Using of transported media for *N. gonorrhoeae* is effective in investigation of gonococcal antimicrobial resistance profile, especially in cases when clinical departments are located distantly from microbiological laboratories. Further expanded evaluation of gonococcal culture in Ukraine is needed, with involving extragenital samples, both from heterosexual and homosexual patients. Implementation of validated and quality-assured culture methods is highly actual and mandatory in Ukraine. The authors declare that they have no competing interest.
